# A mixed-methods community-based participatory research to explore stakeholder’s perspectives and to quantify the effect of crop residue burning on air and human health in Central India: study protocol

**DOI:** 10.1186/s12889-020-09844-6

**Published:** 2020-11-30

**Authors:** Tanwi Trushna, Vishal Diwan, Subroto Shambhu Nandi, Satish Bhagwatrao Aher, Rajnarayan R. Tiwari, Yogesh Damodar Sabde

**Affiliations:** 1 Department of Environmental Health and Epidemiology, ICMR-National Institute for Research in Environmental Health, Bhopal, Madhya Pradesh India; 2 Department of Environmental Monitoring And Exposure Assessment (Water and Soil), ICMR-National Institute for Research in Environmental Health, Bhopal, Madhya Pradesh India; 3 Department of Global Public Health, Karolinska Institutet, Stockholm, Sweden; 4 Department of Environmental Monitoring And Exposure Assessment (Air), ICMR-National Institute for Research in Environmental Health, Bhopal, Madhya Pradesh India; 5 ICMR-National Institute for Research in Environmental Health, Bhopal, Madhya Pradesh India

**Keywords:** Air pollution, Crop residue burning, Community-based participatory research, Focus groups, Key informant interview, India

## Abstract

**Background:**

Crop residue burning adversely affects air quality and consequently human health. India, being one of the largest agro-economies of the world, produces around 500 Million tonnes of crop residue annually most of which is burnt on-farm. However, integrated studies that simultaneously quantify the effects of crop residue burning while exploring the subjective determinants of the practice are lacking in India. This paper describes the protocol for a longitudinal mixed methods research study employing a community-based participatory approach to fill this gap.

**Methods:**

Both quantitative and qualitative data will be collected in a rural setting of the central Indian province of Madhya Pradesh, over 1 year. A steering committee comprising of the research team and community representatives will be formed. The proportion of cultivable land burnt in one crop burning season will be estimated. The association between crop residue burning, level of ambient air pollutants, and pulmonary function of village residents will be determined. Focus groups, interviews, and participatory rural appraisal methods will be used to explore stakeholder perspectives about crop residue burning. Potential barriers and opportunities for substituting burning with an alternative crop residue management technique will be ascertained as the basis for future interventions. Ethics approval has been obtained from the Institutional Ethics Committee of the National Institute for Research in Environmental Health (No: NIREH/BPL/IEC/2019–20/1494, dt 06/01/2020).

**Discussion:**

This manuscript describes the protocol for a novel community-based participatory study to investigate thoroughly the phenomenon of crop residue burning from the perspective of the agricultural community through their active collaboration. The lack of comprehensive evidence regarding the factors responsible for crop residue burning in India underlines the importance of implementing this study protocol to fill in this critical gap in knowledge. While acknowledging that findings of this study will be not generalizable to agricultural communities other than the one studied, it is expected that the study will generate baseline evidence that might be beneficial in developing and implementing an appropriate intervention strategy.

**Supplementary Information:**

The online version contains supplementary material available at 10.1186/s12889-020-09844-6.

## Background

Ambient air pollution (AAP) has emerged as one of the major threats to public health worldwide [1]. According to statistics reported by the World Health Organisation (WHO) in 2018, 4.2 million annual premature deaths occur as a consequence of exposure to AAP [2]. Research has highlighted that exposure to AAP above permissible levels increases morbidity and mortality in humans [3, 4]. Life-long exposure to AAP is the fifth largest risk factor for all-cause mortality and results in a loss of 4.2% of global disability-adjusted life years (DALYs) [5]. Inhalation of ambient air pollutants has been associated with a wide range of morbidities ranging from cardiovascular and respiratory diseases [6, 7] which together account for almost 80% of the global life expectancy loss attributable to air pollution [4] to endocrine [8, 9] and even neuropsychiatric dysfunction [10–12].

Multiple studies have highlighted the adverse impact of agricultural emissions on air quality [13–18]. Burning of crop residues is an important source of agricultural emissions [19] which endangers the environment because of the generation of greenhouse gases and particulate emissions [20–22]. The deliberate use of fire in agriculture is dwindling worldwide but CRB is being increasingly practised in India [23, 24]. India is one of the largest agro-economies of the world [25] and it produces around 500 Million tonnes of crop residue annually [20] most of which is burnt on-farm. According to a recent study, in 2017 CRB resulted in annual emissions of 824 Gg of Particulate Matter (PM_2.5_), 58 Gg of Elemental Carbon, 239 Gg of Organic Carbon, and 211 Tg of greenhouse gases [26]. The authors also predicted that emissions from CRB will increase by 45% in 2050 as compared to 2017 [26].

The negative impact of CRB induced air pollution on human health has been documented by previous research [27, 28]. CRB induces or exacerbates asthma attack [29] and pulmonary function compromise was found to persist even after cessation of burning [30]. Living in areas with high-intensity CRB raises the risk of acute respiratory infection by three times and the danger is most severe in children [31]. Previous studies have demonstrated the presence of carcinogenic benzenoids and other harmful chemicals in emission from CRB [32–34]. Every burning season the number of patients visiting local hospitals in the north Indian province of Punjab rises by almost 10 % [35] and a corresponding increase in average household healthcare expenditure has also been documented [36]. Eliminating emissions from agricultural activities can avert nearly one-fifth of PM_2.5_ related deaths on a global scale [18] and will, therefore, facilitate the achievement of the sustainable development goal (SDG) of substantially reducing mortality and morbidity caused by air, water, and soil pollution by the year 2030[target 3.9 [37]].

The overall public acceptance of government-initiated interventions to contain CRB in India in form of legal enforcement, monetary incentives, as well as penalties, has been poor [38, 39] which is a testament to the complexity of the issue. Even though tackling such a complex issue requires an in-depth understanding of the phenomenon, no research to date has attempted to provide a comprehensive overview of CRB encompassing both quantitative inventories of the effects of CRB as well as an in-depth understanding of the subjective determinants of the practice.

This manuscript thus describes the protocol for an exploratory study to be conducted through the active collaboration of the rural agricultural community in Madhya Pradesh, a province in Central India to collect baseline information using mixed-method approaches. Based on this information, a suitable intervention will be planned and implemented in the subsequent phases to promote the adoption of alternative crop residue management techniques. Specifically, it aims to achieve the following objectives:

### Sub-study 1

1. To estimate using Geographic Information System the proportion of cultivable areas where wheat crop residues are burnt.
2. To evaluate the association between crop residue burning and the concentration of ambient air pollutants.
3. To identify the impact of crop residue burning on the pulmonary function of village residents.

### Sub-study 2

1. To explore the perspective of stakeholders about the effect of crop residue burning on the environment and human health and adoption of potential alternatives.
2. To determine barriers and opportunities for the adoption of alternative crop residue management techniques to identify a suitable intervention for reducing CRB.

## Methods

### Design

**Fig. 1:**
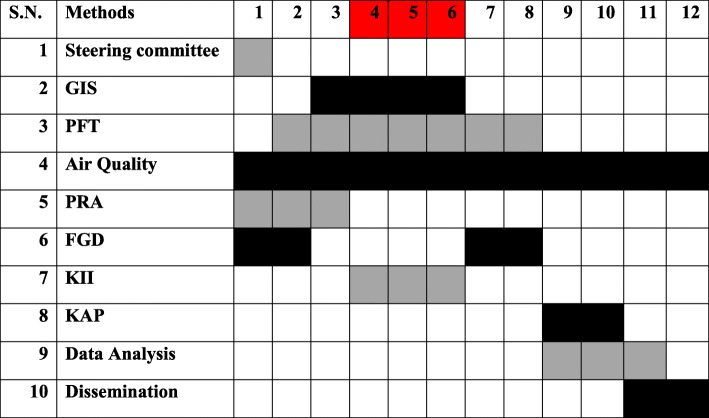
Time Schedule of study methods. Note: The numbered columns represent duration in months (the period of crop residue burning is highlighted in red colour). Abbreviations: FGD: Focus Group Discussion; GIS: Geographic Information system; KAP: Knowledge Attitude Practice; KII: Key Informant Interview; PFT: Pulmonary Function Test; PRA: Participatory Rural Appraisal

This is the protocol for a mixed methods research study that aims to employ a community-based participatory approach to ascertain objective achievement. This study will be conducted over 1 year. The time schedule of various study methods of this protocol is described in Fig. 1.

### Setting

**Fig. 2:**
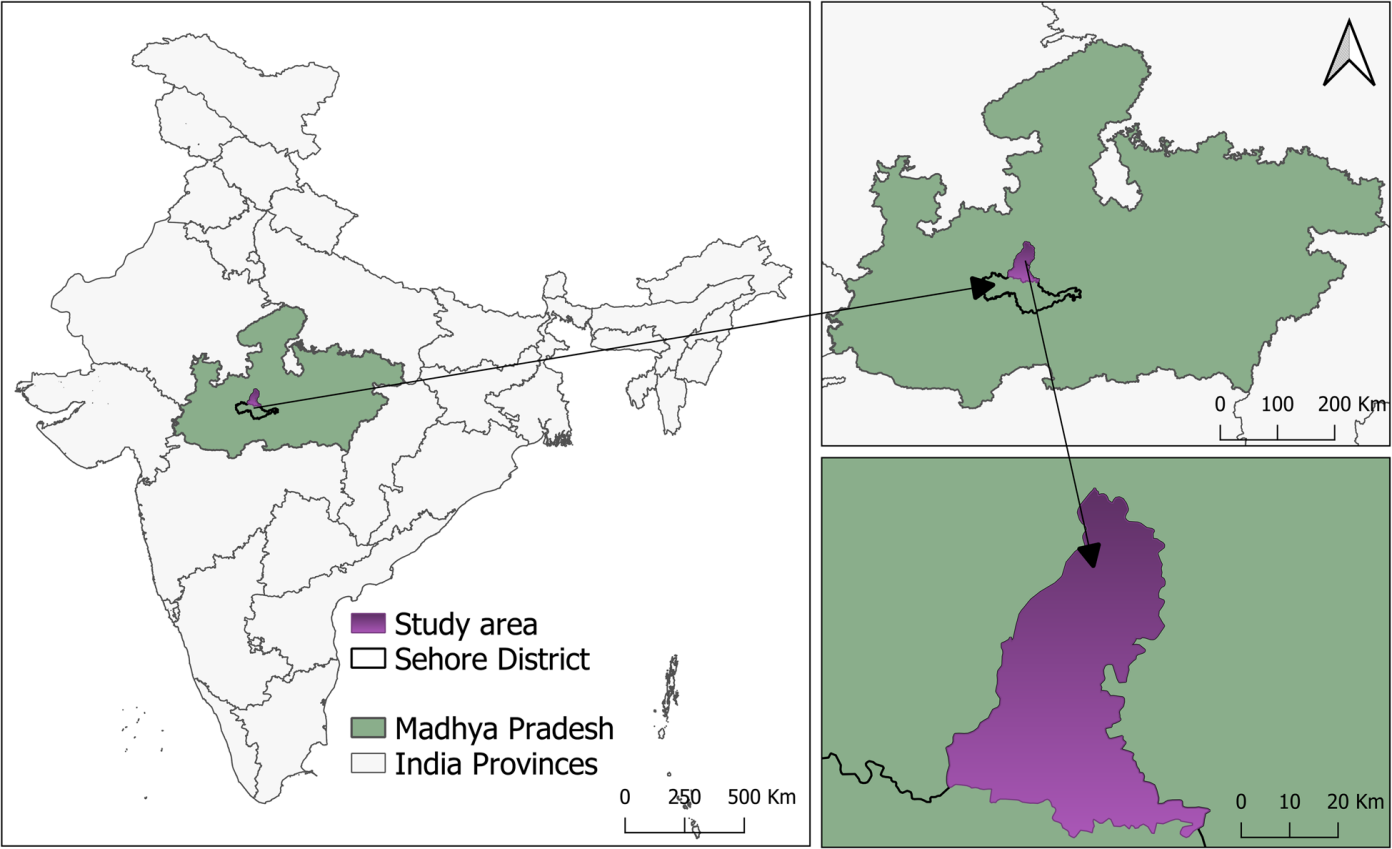
Geographical location of the selected study area. Note: The map shows clockwise, India, Madhya Pradesh, and Sehore District respectively. The village where the study will be conducted will be selected from among the villages of Sehore District. (The map in this figure was created by the authors for use in this manuscript)

This study will be conducted in one administrative subdivision (Shyampur) of Sehore District in the central Indian province of Madhya Pradesh (MP) (Fig. 2).MP has a population of almost 72 million [40], of which almost 75% reside in rural areas and depend on agriculture for their livelihood [41]. Over the last few years, the agricultural growth rate has drastically increased in MP [42]. Thus huge amounts of crop residues (33.18 Million tonnes/year) are being generated and burnt in this province [43]. The predominant cropping pattern in this province involves wheat-soyabean crop rotation [43].

Sehore district consists of eight tehsils (administrative subdivision)covering1031 villages with a total population of 1.3 million [44]. Using the latest national census data [45], we compiled a list of villages of the district in which at least 50% of the total population is involved in agriculture and at least 50% of the total geographic area is used for cultivation. From this list, the village with the largest population and the highest proportion of land under agriculture will be chosen.

### Formation of steering committee

Previous studies adopting a community based participatory approach have engaged their target communities more efficiently through the creation of steering committees comprising of community and academic partners [46–48]. Thus, in the current study, we too will attempt to enhance community participation in research by forming a steering committee consisting of research, technical and community members. The community members of the steering committee who represent the community will act as a bridge for proper communication between researchers and the village community and will help foster a rapport between them.

The committee members will collect sociodemographic and economic information of all village household members for line listing. The steering committee will be involved in planning, feedback, trouble-shooting, and executing the entire study (for example organization of village meetings, participant selection, etc.). They will also be in charge of disseminating the study results to the village community. Committee meetings will be held fortnightly to monitor progress and regularly solve operational issues.

For the selection of community representatives, a village meeting organized will be held on a pre-advertised date. Nominations will be sought for five or more representatives who are knowledgeable about and respected in the community. Community members of the steering committee will represent different socioeconomic classes and will be of both genders.

### Participants and Methods

#### Sub-study 1

##### Objective 1

To estimate using Geographic Information System (GIS) the proportion of cultivable areas where wheat crop residues are burnt.

##### Method

The burden of CRB will be estimated through the calculation of the proportion of total area under cultivation which is burnt during the study period within the administrative boundary of the selected village using the Geographic information system (GIS) and physical field survey. For this, the hard copy of the village administrative boundary maps will be retrieved. These maps will be scanned and registered in the relevant coordinate system for digitization in a vector file using ArcMap 10.7.1. Information regarding burning will be collected by members of the steering committee. The boundaries of farms where crop residues have been burnt as well as the ones where no burning has been done within the administrative limit of the selected village will be plotted as polygons using handheld global positioning system (GPS) survey equipment (GARMIN GPSMAP 64). The data collected will then be exported to GIS as described above for subsequent calculation and data analysis.

##### Objective 2

To evaluate the association between crop residue burning and the concentration of ambient air pollutants.

##### Method

The ambient air quality concerning the key air pollutants viz., PM_10_, PM_2.5_, NO_x,_ and SO_2_ in the vicinity of the identified site will be monitored for 1 year using a respirable dust sampler (RDS)/high volume air sampler (HVAS) placed 3 m above ground in ambient air. The monitoring will be carried out twice a week for 24 h. The sampling time of 4 h and 8 h for particulate pollutant and gaseous pollutants, respectively will be followed as per Ambient Air Quality Monitoring Guidelines issued by the Central Pollution Control Board [49]. PM_10_ and PM_2.5_samples will be collected over previously dried and weighed glass microfibre filters (Whatman GF/A) by drawing the air at a flow rate of 1.1 to 1.2 m^3^ per minute for 8 h. The concentration of particles will be calculated gravimetrically as per standard methods [49].

The presence of SO_2_in ambient air will be detected by absorbing a calculated volume of air into potassium tetrachloromercurate solution which will result in the formation of a stable dichlorosulphitomercurate complex. There afterward, para rosaniline and methyl sulphonic acid will be added and the absorbance of the coloured solution will be detected at 530 nm through the use of a spectrophotometer. Modified West &Gaekemethod will be utilised to calculate the concentration of sulphate ions [49, 50]. Similarly, to measure ambient air NO_2_ a calculated volume of air is passed through sodium hydroxide and sodium arsenite solution. Nitrite ions produced through this process will be determined calorimetrically. Phosphoric acid, sulphanilamide, and N-(1-naphthyl)-ethylenediamine di-hydrochloride will be added to the solution and the absorbance of the coloured azo-dye thus produced will be measured at 540 nm [49, 51, 52].

##### Objective 3

To identify the impact of crop residue burning on the pulmonary function of village residents.

##### Participants

Healthy [non-smoker; no recent (within previous 1 month) history of myocardial infarction, aneurysm, surgery; no active diseases (e.g. pulmonary Tuberculosis, active haemoptysis, orofacial pain, acute respiratory infection, etc.); no pregnancy] adults (≥18 years of age) and adolescents (6 to < 18 years of age) of both genders.

##### Method

The number of participants to be sampled was calculated using the nMaster 2.0 software based on the differences in lung function (Forced vital capacity) before and after wheat crop residue burning as previously reported by a study conducted in Punjab, India by Awasthi et al., 2010 [53]. A sample size of 105 participants provides a 90% power to detect a measurable difference in lung function before and after exposure to CRB with a two-sided type I error of 5%, assuming that the non-responder rate will be 10%. Thus pulmonary function testing will be done in 105 adults and 105 children.

Systematic random sampling will be used for the selection of participants. From a list of all village households, 105 households will be randomly chosen. From the selected households, one eligible adult and one child participant of either gender will be selected. If there are > 1 eligible participants in the household, then one will be selected using the Troldahl-Carter-Bryant Respondent Selection Method [54] (Table 1).

**Table 1:** Schema for the selection of eligible individual from the household

Number of adult/child females in the household	Number of adults/children in the household			
	1	2	3	4 or more
**0**	Male	Youngest Male	Youngest Male	Oldest Male
**1**	Female	Female	Oldest Male	Female
**2**		Oldest Female	Male	Oldest Male
**3**			Youngest Female	Oldest Male
**4 or more**				Oldest Female

Pulmonary function test (PFT) will be done monthly- once before, during (roughly 3 months- three times), and once after the last field is burnt. The age, height, and weight of all participants will be recorded on the first contact. Each participant will be asked to fill a questionnaire to elicit data regarding other risk factors that can affect PFT such as occupational details, medical history, etc. The investigation will be conducted using a portable spirometer (EasyOne Model no. 2001) on comfortably seated participants.

PFT parameters like forced expiratory volume in 1st second (FEV_1_), forced vital capacity (FVC), and peak expiratory flow (PEF) will be assessed through spirometry administered by a trained technician, supervised by a trained physician, following national guidelines for spirometry [55]. At least three acceptable spirograms will be obtained such that the two largest FVC measurements and the two largest FEV1 measurements vary by ≤0.150 l. Testing will be repeated either until the criteria are fulfilled or until the test has been performed eight times. The entire procedure will require around 20–30 min. The best measurement for each parameter will be recorded for each participant.

#### Sub-study 2

##### Objective 1

To explore the perspective of stakeholders about the effect of crop residue burning on the environment and human health and the adoption of potential alternatives.

##### Objective 2

To determine barriers and opportunities for the adoption of alternative crop residue management techniques to identify a suitable intervention for reducing CRB.

The aforementioned objectives will be achieved through qualitative research methods enlisted below (Details in Table 2):

1. *Participatory rural appraisal*

**Table 2:** Details of PRA tools to be used, participants and data to collected using them

Tool	Information to be collected using it	Participant groups
Objective 1 of sub-study 2:		
**Qualitative Research Methods:**		
FGD	To explore participants’ views regarding CRB, its adverse consequences, and their reasons for continuing CRB.	Village residents
KII	To elicit stakeholders’ perspectives about CRB and its effects specific to their area of expertise (for example, local health care providers will be asked to describe effects of CRB on the health profile of village residents, etc.)	Stakeholders indirectly involved with CRB
**PRA tools:**		
Trend Analysis [56]	To understand the historical chronology of crop residue management practices adopted by the villagers with a special focus on CRB and to generate discussions regarding any perceived association between the health status of villagers and the onset of CRB.	Elderly farmers
Seasonal Analysis [57]	To explore the association of perceived air quality and health concerns with CRB season	Adults with chronic respiratory diseases, Mothers with young children, Health care workers residing in the village.
Scoring Method [58]	To understand the community’s perception regarding air quality concerns (including CRB) and related health consequences in the village	Male and female adults according to residential proximity to farmland
Objective 2 of sub-study2:		
**Qualitative Research Methods:**		
FGD	To explore participants’ opinions and concerns/ perceived barriers to adopting an alternative crop residue management practice and their level of interest in supporting or opposing an intervention aimed at changing the current crop residue management practice.	Village residents
KII	To discuss their perspectives about potential barriers and opportunities along with their level of interest in supporting the implementation of an intervention aimed at promoting the adoption of healthy crop residue management techniques.	Stakeholders indirectly involved with CRB
**PRA tools:**		
Resource Mapping [57]	To identify the potential resources in the village that can influence the planning of an alternative crop residue management practice. For example, participants will be asked to depict the way their village looks like while focussing on the various resources pertinent to farming such as the spatial distribution of farmlands, water reservoirs, storage spaces, dairy farms (if any), etc.	Village leaders, self-help groups e.g. agriculture groups
Social Mapping [57]	To explore the habitation pattern in the village especially concerning the farms to understand the probable effect of CRB on human health To understand the social infrastructure and the inherent hierarchy in the village community which can have a bearing on the individual decision-making process of the villagers and thus on the acceptance of an intervention	Male and female representatives from different social categories e.g. castes
Wealth Ranking [58]	To understand the local perceptions regarding wealth distribution in the village and thus the economic hierarchy in the village community which can have a bearing on the individual decision-making process of the villagers and thus on the acceptance of an intervention	Male and female representatives from different socioeconomic categories e.g. landowners vs landless labourers

*Abbreviations*: *CRB* Crop Residue Burning, *FGD* Focus Group Discussion, *KII* Key Informant Interview, *PRA* Participatory Rural Appraisal

##### Participants

Village community members.

##### Method

Participants will be selected through purposive sampling by community members of the steering committee utilizing their “insider” knowledge of the community. A combination of six PRA tools, namely resource mapping, social mapping, wealth ranking, trend analysis, seasonal analysis, and scoring method will be used to understand the community’s perception of CRB, its effect on the environment and human health, and to explore potential barriers and opportunities to the adoption of alternative crop residue management techniques (Table 2).

Village meetings will be organized and PRA activities will be held as per standard guidelines [59]. Moderator will facilitate the process and the note-taker will actively take notes. Participants will be asked to pictorially depict their perspectives for mapping activities and to provide relative ranks and scores for the rest methods. Researchers will probe to clarify aspects that are not clear. Local materials such as stones, leaves, trees sticks, etc. will be were used in addition to flip charts, marker pens, maps, GPS. At the end of the activity, the depicted maps will be saved (either copied onto papers or photographs of for maps created on ground capture using cameras) for analysis.

2. *Focus Group Discussion*

##### Participants

Village residents.

##### Methods

Participants will be selected through a purposeful sampling method with maximum variation in age, gender, and land ownership and FGDs will be continued until saturation is attained [60, 61]. Using a semi-structured topic guide (Appendix 1 in Supplementary Material), researchers will attempt to elicit participants’ views regarding CRB, its adverse consequences, and their reasons for continuing CRB. Their opinion and concerns/ perceived barriers to adopting an alternative crop residue management practice will also be explored. We will further explore the participants’ level of interest in supporting or opposing an intervention aimed at changing the current crop residue management practice. The resources at their disposal, their alliances, and their level of influence on the village community will also be discussed.

3. *Key Informant Interview:*

##### Participants

Stakeholders indirectly involved with CRB such as local health care providers, agriculture extension workers, employees of government and non-government organizations, local political leaders, etc.

##### Methods

Participant selection will be done through purposive sampling and interviews will be continued until saturation is attained [60, 61]. Participants’ perspectives about CRB and its effects specific to their area of expertise (for example, local health care providers will be asked to describe effects of CRB on the health profile of village residents, etc.) will be elicited using a topic guide (Appendix 2 in Supplementary Material). Their perspectives about potential barriers and opportunities along with their level of interest in supporting the implementation of an intervention aimed at promoting the adoption of healthy crop residue management techniques will also be discussed.

The discussion and interviews will be conducted in the local language (Hindi) by research staff trained in qualitative data collection methods [62, 63]. The duration of each discussion/interview will be approximately 60–90 min. Audio-recording will be done and field notes will be taken.

### Quality control

Various control measures will be adopted to ensure the collection of high-quality data. Necessary training will be research staff. Quantitative and qualitative data collection tools will be validated before use. A pilot study in a nearby rural setting will be conducted to field test the study methodology, data collection instruments and to train the research assistants. Similarly, the quality of air pollutant concentration estimation will be ensured through meticulous checking of filter papers, sampling equipment, and reagents to be used.

### Data management

The investigator assigned the responsibility of data management will ensure that the data is stored safely and timely back-ups are maintained.

#### Quantitative Data management

Filled questionnaires and samples will be assigned unique identifiers. Data from questionnaires and those generated during the analysis of laboratory samples will be entered into the latest version of Microsoft Excel spreadsheets. Data from all sub-studies will be linked. Senior researchers will supervise data entry and double-check at least 10 % of the entered data for quality assurance. Post data entry, hard copies of questionnaires will be stored in a secure archive in the institute. All computer-based data kept under password protection will be handled by research staff alone.

#### Qualitative Data management

Hard copies of maps and ranking charts generated during PRA activities will be converted into soft copies containing relevant identifying details such as date, title, and purpose of the exercise. These hard copies along with the field notes of FGDs and KIIs will be stored in a secure archive in the institute whereas soft copies and audio-recordings will be stored confidentially in password-protected computers.

### Data analysis

#### Data analysis of quantitative data (sub-study 1)

Analysis of the data will be done using SPSS statistics software (Version 26). Descriptive statistics like mean, median, standard deviation, 95% confidence interval, interquartile range, and percentages/proportion will be used. The total cultivable area and the area where crop residues are burnt in the village will be calculated using the area calculator tool of ArcMap (10.7.1). The proportion of area burnt out of the total cultivable area will then be estimated. The timing and proportion of cultivable area burnt will then be correlated with the trend of change in the concentration of air pollutants recorded, The mean change in the PFT parameters of the villagers before, during, and after CRB will be calculated. ANOVA repeated measures will be used to check for any significant change in the PFT parameters across different time points. Multiple linear regression analysis will be used to detect the independent effect of CRB on PFT parameters while adjusting for known confounders.

#### Data analysis of qualitative data (sub-study 2)

Audio-recording and field notes will be used to create transcripts and translation of those into English. Thematic analysis approach using the Framework Method [64] will be used to analyse the data collected. Data coding and analysis will be done by two researchers separately (TT and VD) and interpretations will then be compared. In the case of conflict, the resolution will be done through a consensus reached by all committee members.

**Fig. 3:**
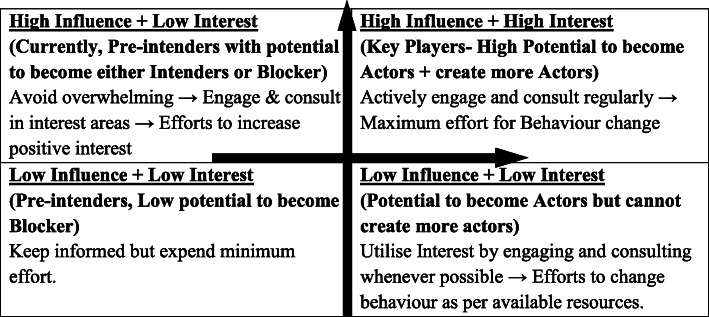
Influence-Interest Grid. Note: Influence is plotted on the vertical axis and Interest on the horizontal axis. According to the details plotted on this grid, a stakeholder management plan will be devised for behaviour change intervention in the next phase of the study

Stakeholder mapping: Based on the themes generated from analysis and the information obtained during social mapping & wealth ranking, stakeholder mapping will be done following the Power-interest grid as shown in Fig. 3 [65]. The presence of influence directions and the strength of influence will also be noted wherever possible.

### Dissemination

Dissemination of the study findings will be done in two stages. In the first stage, with the help of the community members of the steering committee, village meetings will be organised to inform the participating community regarding the conclusions drawn. After appropriate discussion with the community, in the second stage, dissemination will be done in scientific forums through publications.

### Ethical considerations

Ethics approval has been obtained from the Institutional (Human) Ethics Committee, National Institute for Research in Environmental Health (No: NIREH/BPL/IEC/2019–20/1494, dt 06/01/2020). Before the initiation of research activities, the community of the selected village will be approached starting with the gatekeepers (village leaders) whose consent will be solicited for the creation of the steering committee.

Before the onset of data collection, each participant (guardians for PFT participants< 18 years of age) will be provided detailed oral and written information in Hindi (local language) and English regarding the aim and procedure of study in an easy to understand manner and all queries will be answered. After reiterating the fact that participation is voluntary and that the information shared will be kept confidential, participants will be asked to sign the consent form (Appendix 3,4 in Supplementary material). In case the participant is below 18 years of age, parental/guardian written consent will be obtained along with assent from the child (Appendix 5 in Supplementary material). In addition, permission for audio-recording of interviews and focus group discussions will be obtained. Necessary precautions (e.g. use of disposable mouthpieces) for prevention of the spread of airborne infections will be applied while performing spirometry. In case of any discomfort experienced by the participants during the procedure, doctors in the research team will assess the participant and will refer those requiring medical attention to the nearest health-care facility.

## Discussion

This protocol describes a mixed-methods research study that will be conducted in the rural setting of the central Indian province of Madhya Pradesh to study the diverse and complex problem of CRB. The burden of CRB in terms of the proportion of cultivable areas where wheat crop residues are burnt will be estimated using field surveys, GPS, and GIS. Many previous studies have employed GIS in conjunction with satellite data to estimate the CRB burden [66, 67]. However, in the current study protocol CRB burden will be estimated for one village and thus can be easily done even without the use of remote sensing data.

Previous studies in India have documented the emission of air pollutants owing to CRB [68, 69] and the consequent effect on the pulmonary health of exposed individuals [31, 70]. However, most of these previous studies have mostly focussed on the northern parts of India especially Punjab and Haryana. The current study protocol aims to document the effect of CRB on air quality and human health in Madhya Pradesh, a province in central India. Over the last few years, a drastic increase has been noted in the agricultural growth rate in MP leading to higher production of grains and thus residues [71]. As a result, CRB is no longer restricted to the traditional pre-winter burning of paddy residues in northern India and instead has spread to MP as well [72–74]. Nevertheless, not many studies have documented the extent and effect of CRB in MP. The current protocol when implemented will fill this critical gap in information.

Few studies have attempted to scientifically explore the reasons behind the widespread persistent acceptance of CRB by farmers in India despite contrary legal enforcement [75–79]. But even those studies have mostly used structured questionnaires to quantitatively compile the reasons [76–78] and only a couple of studies have qualitatively explored farmers’ perspectives [75, 79]. A mixed-methods approach will thus be adopted in the current study to overcome this lacuna.

CRB is a multidimensional issue since it affects and is in turn affected by other critical sustainability issues confronting agriculture such as groundwater depletion and thus the generation of a solution requires multi-disciplinary research focussing on the multitude of related aspects of CRB [80]. Therefore, the current protocol thus chooses to focus on multiple objectives with an overarching goal to document baseline evidence to assist in the development of a suitable intervention designed to promote the adoption of an alternative crop residue management technique. Interventions to curb CRB have not been initiated in MP and even those that have been implemented elsewhere have not been widely successful [38, 39]. In this context, it is important to note that CRB, although harmful to the environment in large [68, 69], does not produce immediate clinically apparent individual-level adverse health effects and instead is inexpensive [81] and even appears to increase the yields of next sown crop [82]. Thus, it is a behaviour widely adopted by farmers [81, 83]. To promote an alternative to such an ingrained and apparently beneficial behaviour, the implemented intervention will need to overcome the inertia and hesitation to change [84]. Research has highlighted the role played by theory-driven behaviour change interventions in promoting sustainable pro-environment change [85, 86]. The baseline data collected through the current study will thus enable planning, implementation, and evaluation of an appropriate intervention to curb CRB in MP.

Public acceptance of any intervention is crucial to its success [87]. The benefits of using a community-based participatory approach in this context are gradually being recognised in public health research since it increases collaboration between academia and the community [88, 89]. Partnering with the stakeholder community allows for the research findings to be grounded in the context, enriched by diverse perspectives and expertise, and therefore more appropriate and acceptable to the community [90]. Thus the current study protocol, through the formation of a steering committee consisting of community representatives as members will attempt to ensure collaboration and foster mutual trust and rapport which in turn will facilitate our understanding of the hitherto unexplored perspectives of stakeholders. The use of participatory techniques such as PRA and focus groups will empower the community members to actively engage in discussing problems as well as identifying appropriate and acceptable solutions [91, 92].

### Methodological considerations

To the best of our knowledge, the current study is the first to employ a participatory approach to collaborate with the agricultural community in analysing the extent and effect of CRB. The juxtaposition of qualitative and quantitative research methods for data collection in the current study will yield comprehensive information regarding CRB and this will be actualised through a multi-disciplinary research team that constitutes a potential strength of this study. Furthermore, the use of varied participant groups, multiple data sources, and collection methods, as well as several researchers in a study, through a data source, method, and investigator triangulation [93], will augment the credibility and validity of the study results.

This study protocol will be implemented in a limited geographical area and the findings of this study will be specific to the studied population. Thus insights generated cannot be accurately extrapolated to diverse populations. However, lessons learnt during this study will aid the conduct of similar research in other communities. Furthermore, no control group will be included in this study which reduces our ability to compare and derive accurate conclusions regarding the effect of CRB on air quality. Despite the fact that participants will simultaneously be exposed to pollutants in their home environment, we will not be able to monitor and measure indoor air pollution.

## Conclusion

This manuscript describes the protocol for a novel community-based participatory study to investigate thoroughly the phenomenon of CRB from the perspective of the agricultural community through their active collaboration. The lack of comprehensive evidence regarding the factors responsible for CRB, especially in MP underlines the importance of implementing this study protocol to fill in this critical gap in knowledge. While acknowledging that findings of this study will be not generalizable to agricultural communities other than the one studied, it is expected that the study will generate baseline evidence that might be beneficial in developing and implementing an appropriate intervention strategy to mitigate CRB in MP.

## Supplementary Information

**Additional file 1. **Supplementary Material **Appendix 1.** Focus Group Discussion (FGD) Tentative Topic Guide- Village Residents. **Appendix 2.** Key Informant Interview (KII) Tentative Topic Guide- Stakeholders. **Appendix 3.** Patient Information Sheet and Consent form- Qualitative data collection. **Appendix 4.** Patient Information Sheet and Consent form- PFT (adults). **Appendix 5.** Patient Information Sheet, Parental Consent and Child Assent form- PFT (Child).

## Data Availability

Not applicable (Data sharing does not apply to this article which describes a study protocol and thus no datasets have been generated or analysed yet).

## Abbreviations

AAP: Ambient air pollution
CRB: Crop residue burning
DALY: Disability-adjusted life years
FEV_1_: Forced expiratory volume in 1st second
FGD: Focus Group Discussion
FVC: Forced vital capacity
GIS: Geographic Information system
HVAS: High volume air sampler
KAP: Knowledge Attitude Practice
KII: Key Informant Interview
MP: Madhya Pradesh
NO_x_: Nitrogen oxides
PEF: Peak expiratory flow
PFT: Pulmonary Function Test
PRA: Participatory Rural Appraisal
PM_10_: Particulate matter with aerodynamic diameter < 10 μm
PM_2.5_: Particulate matter with aerodynamic diameter < 2.5 μm
RDS: Respirable dust sampler
SO_2_: Sulphur dioxide
SDG: Sustainable Development Goal
WHO: World Health Organisation
m: Metre
cm: Centimetre (1 cm = 10^− 2^ m)
mm: Millimetre (1 mm = 10^− 3^ m)
nm: Nanometre (1 nm = 10^− 9^ m)
g: Gram (1 g = 10^− 3^Kilogram)
Gg: Gigagram (1Gg = 10^6^Kilogram)
Tg: Teragrams (1Tg = 10^9^Kilogram)
ml: Millilitre (1 ml = 10^− 3^ l)
M: Molar mass of the substance (the mass of one mole of the substance) in g/mol
N: Gram equivalent weight of a solute per litre of solution
