# Supplementary material for: A mixed-methods community-based participatory research to explore stakeholder’s perspectives and to quantify the effect of crop residue burning on air and human health in Central India: study protocol
Source: BMC Public Health. 2020 Nov 30;20:1824. doi: 10.1186/s12889-020-09844-6 (PMC7706198; doi:10.1186/s12889-020-09844-6)
Supplement: Supplementary file 1 — Additional file 1. Supplementary Material Appendix 1. Focus Group Discussion (FGD) Tentative Topic Guide- Village Residents. Appendix 2. Key Informant Interview (KII) Tentative Topic Guide- Stakeholders. Appendix 3. Patient Information Sheet and Consent form- Qualitative data collection. Appendix 4. Patient Information Sheet and Consent form- PFT (adults). Appendix 5. Patient Information Sheet, Parental Consent and Child Assent form- PFT (Child). [file 12889_2020_9844_MOESM1_ESM.docx]

**Supplementary Material**

**Table of Contents:**

1. Appendix 1 [Focus Group Discussion (FGD) Tentative Topic Guide]
2. Appendix 2 [Key Informant Interview (KII) Tentative Topic Guide]
3. Appendix 3 [Patient Information Sheet and Consent form- Qualitative data collection]
4. Appendix 4 [Patient Information Sheet and Consent form-PFT (adults)]
5. Appendix 5 [Patient Information Sheet, Parental Consent and Child Assent form- PFT (Child)]

**Appendix 1: Focus Group Discussion (FGD) Tentative Topic Guide- Village Residents**

**INSTRUCTIONS to the Facilitator:** Before the start of the topic-related discussion, introduce yourself and other team members (if any), clearly state the reason for conducting the FGD, and finally establish the ground rules.

Questions posed to the participants will be of two levels:

• **Primary discussion questions (bold text)**: These questions are pertinent to the major areas of discussion based on the study objectives and have to be discussed with the participants by the end of the FGD. The questions written in this topic guide serve as the basis for the initiation of discussion and to ensure the consistency of questions asked across FGDs. But the facilitator is free to adapt the questions/ their order based on the ongoing discussion.

• **Probing questions: (text in Italics):** These points can be helpful to promote further discussion and elicit information from the participants when their responses to the primary question are too brief. Thus based on what has already been covered in the discussion and the flow of the ongoing discussion, the facilitator can introduce these probing questions as and when needed.

Start of audio-recording

Greetings! I am _ and I have with me here my team member(s) _. As discussed with you (during the consent process), we are speaking with individuals who have personal experience or knowledge regarding crop residue burning (CRB) as part of our research study which aims to understand the phenomenon from the perspective of people who are in one way or the other directly involved in it. This group discussion is a part of that project. We will today discuss your knowledge regarding CRB, your perception of its effect on humans, and your opinions regarding the need for change.

Before I begin I want to request every participant to take part in the discussion. However, kindly speak one at a time. If someone is speaking, please wait till he/she is done. There is no specific order of speaking to follow. There are no right or wrong answers and everyone is entitled to their viewpoints. Also, what is being discussed here will be kept confidential. The discussion is being audio-recorded and so I request for mobile phones to be kept on silent/vibration mode.

I will start when you are ready and any doubts that you have, have been cleared. Does anyone have any questions? (Wait for responses) Ok! Let’s begin.

I would like to start our discussion by knowing more about you. Kindly tell us about yourself.

(Encourage to describe themselves, their social position in the community, economic status, and their leadership experience if any)

| **Sl.No.** | **Discussion Points** |
| --- | --- |
| 1 | Crop residue management in the village |
| **a)** | **Can you tell me how crop residues left in the farmland after harvesting are managed in your village?** |
|  | Possible Probes:   - *Attitude towards CRB* - *Knowledge and attitude towards other crop residue management techniques* - *How common is CRB as compared to other crop residue management techniques?* |
| **b)** | **Now that you have described how crop residues are currently managed in your village, I am curious to understand how it was done in the earlier days.** |
|  | Possible Probes:   - *History of CRB* - *If there was a change in crop residue management, what triggered the change?* |
| 2 | Effect of different crop residue management techniques on the environment and human health |
| **a)** | **How do you think the crop residue management techniques used in your village might affect the environment around you?** |
|  | Possible Probes:   - *Effect of CRB on air quality, soil and underground water* - *Difference between the effect of CRB and other crop residue management techniques on-air/soil/water.* |
| **b)** | **How do you think the crop residue management techniques used in your village might affect the health of village residents?** |
|  | Possible Probes:   - *Effect of CRB on (pulmonary) health of the vulnerable population (e.g. young children, pregnant women, elderly, people with chronic respiratory diseases like asthma, etc.)* - *Difference between the effect of CRB and other crop residue management techniques on health* - *The health of the vulnerable population in earlier days when CRB was not so commonly used (depending upon the discussion of point 2b)* |
| 3 | Need for continuing CRB versus need for change |
| **a)** | **There has been a lot of debate on continuing CRB. There have been many government initiatives to stop CRB. Why do you think farmers in general (including yourself and others in your village) burn crop residues?** |
|  | Possible Probes:   - *What advantages are offered by CRB* over *alternative crop residue management techniques (e.g. saving time/money)?* - *Influence of other people’s decisions on participants (i.e. participants themselves burn crop residues because that is what everyone/people they trust/respect does)* - *Inertia to change something that is known and fear of adopting a new/unknown method* |
| **b)** | **What is your opinion on the need to reduce CRB and switch to alternative crop residue management techniques?** |
|  | Possible Probes:   - *What are the anticipated problems if farmers switch to alternative crop residue management techniques?* - *Technical know-how/Skill to use alternative crop residue management techniques* - *Economical- Perspective about change in expenditure* - *Social/ Psychological – Concerns about perceived peer pressure/ change in judgement by others in their social circle, Fear of the unknown?* - *What will be the potential advantages (e.g. fodder for cattle, soil fertility, less air pollution, less effect on human health) if farmers switch to alternative crop residue management techniques?* |
| 4 | Way of change |
| **a)** | **If an intervention is designed and implemented to stop CRB and promote the adoption of an alternative crop residue management technique, how will you and others (not present here) in your village react?** |
|  | Possible Probes:   - *Attitude towards the intervention (Support/oppose, level of interest, intent to actively participate, thoughts on success/failure)* - *If enthusiastic about intervention- How can they contribute (e.g. influence others etc.)?* - *How will their stand affect those close to them (assess the strength of their alliances and the potential influence they might have over other stakeholders)?* - *The perceived reaction of individuals of the village (not present in the current FGD) who are major influencers? (Who among them are likely to have a favourable opinion about the intervention and who are likely to oppose? How strongly do you think they feel about CRB and an intervention to mitigate it?)* |
| **b)** | **What would be your suggestions for an intervention that you believe would be useful?** |
|  | Possible Probes:   - *Level of intervention- government level/policy level changes or community level changes* - *Content of intervention- IEC/financial assistance* - *Whom should it target for maximum impact* - *When and how should it be launched?* |

Please feel free to continue if you have more to add to this discussion.

Many thanks for your participation. This has been a very interesting discussion and we hope all of you think so as well. The opinions and perspective expressed here will enrich our study and I would like to repeat that all comments will be kept confidential and anonymous.

If you wish to express anything else, kindly contact the phone numbers mentioned in the information sheet provided to you earlier. Before you leave, please hand in the completed personal details questionnaire.

Name of Moderator: __________________________

Signature: __________________________

FGD Number: __________________________

Date: __________________________

**Appendix 2: Key Informant Interview (KII) Tentative Topic Guide- Stakeholders**

**INSTRUCTIONS to the Facilitator:** Before the start of the topic-related discussion, introduce yourself and other team members (if any), clearly state the reason for conducting the KII, and finally establish the ground rules.

Questions posed to the participants will be of two levels:

• **Primary discussion questions (bold text)**: These questions are pertinent to the major areas of discussion based on the study objectives and have to be discussed with the participants by the end of the KII. The questions written in this topic guide serve as the basis for the initiation of discussion and to ensure the consistency of questions asked across KIIs. But the facilitator is free to adapt the questions/ their order based on the ongoing discussion.

• **Probing questions: (text in Italics):** These points can be helpful to promote further discussion and elicit information from the participants when their responses to the primary question are too brief. Thus based on what has already been covered in the discussion and the flow of the ongoing discussion, the facilitator can introduce these probing questions as and when needed.

Start of audio-recording

Greetings! I am _ and I have with me here my team member(s) _. As discussed with you (during the consent process), we are speaking with individuals indirectly involved with CRB such as local health care providers, agriculture extension workers, employees of government and non-government organizations, local political leaders, etc. This interview is a part of that project. We will today discuss your perspectives about CRB and its effects specific to your area of expertise and your opinions regarding the need for change.

Before I begin I want to request you to actively take part in the discussion as your perspective will enrich our study data. There are no right or wrong answers and what is being discussed here will be kept confidential. The discussion is being audio-recorded and so may I request you to kindly keep your mobile phone(s) on silent/vibration mode, if possible?

I will start when you are ready and any doubts that you have, have been cleared. Do you have any questions? (Wait for response) Ok! Let’s begin.

I would like to start our discussion by knowing more about you. Kindly tell us about yourself.

(Encourage to describe himself/herself, their social position in the community, economic status, and their leadership experience if any)

| **Sl.No.** | **Discussion Points** |
| --- | --- |
| 1 | Crop residue management in the village |
|  | **You are associated with _ village in your work capacity as _. Can you tell me how crop residues left in the farmland after harvesting are managed in this particular village (from what you have seen)?** |
|  | Possible Probes:   - *Attitude towards CRB* - *Knowledge and attitude towards other crop residue management techniques* - *How common is CRB as compared to other crop residue management techniques?* |
| 2 | Effect of different crop residue management techniques on the environment and human health |
| **a)** | **How do you think the crop residue management techniques used in this village might affect the environment?** |
|  | Possible Probes:   - *Effect of CRB on air quality, soil and underground water* - *Difference between the effect of CRB and other crop residue management techniques on-air/soil/water.* |
| **b)** | **How do you think the crop residue management techniques used in this village might affect the health of village residents?** |
|  | Possible Probes:   - *Effect of CRB on (pulmonary) health of the vulnerable population (e.g. young children, pregnant women, elderly, people with chronic respiratory diseases like asthma, etc.)* - *Difference between the effect of CRB and other crop residue management techniques on health* - *The health of the vulnerable population in earlier days when CRB was not so commonly used (depending upon the discussion of point 2b)* |
| 3 | Need for continuing CRB versus need for change |
| **a)** | **There has been a lot of debate on continuing CRB. There have been many government initiatives to stop CRB. Why do you think farmers in this village burn crop residues?** |
|  | Possible Probes:   - *What advantages are offered by CRB* over *alternative crop residue management techniques (e.g. saving time/money)?* - *Influence of other people’s decisions on participants (i.e. participants themselves burn crop residues because that is what everyone/people they trust/respect does)* - *Inertia to change something that is known and fear of adopting a new/unknown method* |
| **b)** | **What is your opinion on the need to reduce CRB and switch to alternative crop residue management techniques?** |
|  | Possible Probes:   - *What are the anticipated problems if farmers switch to alternative crop residue management techniques?* - *Technical know-how/Skill to use alternative crop residue management techniques* - *Economical- Perspective about change in expenditure* - *Social/ Psychological – Concerns about perceived peer pressure/ change in judgement by others in their social circle, Fear of the unknown?* - *What will be the potential advantages (e.g. fodder for cattle, soil fertility, less air pollution, less effect on human health) if farmers switch to alternative crop residue management techniques?* |
| 4 | Way of change |
| **a)** | **If an intervention is designed and implemented to stop CRB and promote the adoption of an alternative crop residue management technique, how will you and others (not present here) in your work area react?** |
|  | Possible Probes:   - *Attitude towards the intervention (Support/oppose, level of interest, intent to actively participate, thoughts on success/failure)* - *If enthusiastic about intervention- How can they contribute (e.g. influence others etc.)?* - *How will their stand affect village residents (assess the strength of their alliances and the potential influence they might have over other stakeholders)?* - *The perceived reaction of individuals of the village (not present in the current FGD) who are major influencers? (Who among them are likely to have a favourable opinion about the intervention and who are likely to oppose? How strongly do you think they feel about CRB and an intervention to mitigate it?)* |
| **b)** | **What would be your suggestions for an intervention that you believe would be useful?** |
|  | Possible Probes:   - *Level of intervention- government level/policy level/community level changes* - *Content of intervention- Information Education Communication campaigns /financial assistance* - *Whom should it target for maximum impact?* - *When and how should it be launched?* |

Please feel free to continue if you have more to add to this discussion.

Many thanks for your participation. This has been a very interesting discussion and we hope all of you think so as well. The opinions and perspective expressed here will enrich our study and I would like to repeat that all comments will be kept confidential and anonymous.

If you wish to express anything else, kindly contact the phone numbers mentioned in the information sheet provided to you earlier. Before you leave, please hand in the completed personal details questionnaire.

Name of Moderator: __________________________

Signature: __________________________

FGD Number: __________________________

Date: __________________________

Note: This is a tentative topic guide. Since we will be approaching diverse stakeholder groups such as local health care providers, agriculture extension workers, employees of government and non-government organizations, local political leaders, etc., we will adapt the questions according to their area of expertise and/or interest.

**Appendix 3: Patient Information Sheet and Consent form- Qualitative data collection**

**INFORMED CONSENT FORM**

This informed consent has two parts:

Part-1: Information Sheet (To share information about the study with you)

Part-2: Certificate of Consent (For signature if you agree to take part)

**PART-1**

**(PARTICIPANT INFORMATION SHEET)**

**Title of this study: Community-based participatory action research for situation analysis and mitigation of crop residue burning in Madhya Pradesh, India.**

**Name of Principal Investigator:** Dr. Tanwi Trushna, Scientist-B, Epidemiology, ICMR-NIREH, Bhopal

**Co-Investigators:** Dr. Yogesh Sabde, Dr. Vishal Diwan, Dr. Subroto Nandi, Dr. Satish B. Aher.

**Introduction:**

You are invited to take part in this study. The title of this study is “Community-based participatory action research for situation analysis and mitigation of crop residue burning in Madhya Pradesh, India”. The purpose of this form is to provide you with information regarding this research project and if you agree to sign the consent form, then it means that you are willing to participate. It is very important that you carefully read and understand the information provided in this form. This form describes in detail the purpose, procedure, benefits, and risks of the current study. There might be words or processes that you might not be able to understand and in that case, you are encouraged to ask questions to clarify your doubts and the investigators would be willing to answer them. You are at complete liberty to choose to participate in the study or withdraw your participation at any point in time.

**Purpose of the study:** The current study aims to comprehensively understand the various aspects of crop residue burning from the perspective of the community and to estimate the impact of the same on human health, air quality, and soil. The study will attempt to identify the barriers to the adoption of healthy crop residue management behaviour and possible strategies for behaviour change intervention to promote the same.

**Study Procedures:** Presently I will explain in detail all the procedures and in case, you need extra information, please feel free to ask at any time. No procedure will be initiated before you provide consent to participate in the study. You do not have to give your consent or answer any question if you do not feel comfortable due to any reason. The information that you choose to share with the investigators will be completely confidential and your identity will never be disclosed.

**Details of the procedure:** Investigators will collect information regarding crop residue burning from you. You will be asked to discuss your perceptions regarding the effects of crop residue burning on human health, air quality, and soil fertility. Also, your opinion on the possible alternatives to crop residue burning will be solicited in the interview/focus group discussion/Participatory rural appraisal (PRA) activity. The process of such qualitative data collection will happen over one year during which you will be expected to participating in interviews/focus group discussions/PRA activities.

**Potential risks or side-effects:** None

**Potential benefits:**

Immediate benefits: The point of view of the agricultural community which has been ignored to date will be understood by scientists and through joint participation, an effective and sustainable solution for the issue of crop residue burning might be identified.

Delayed benefits: Better health and a smoke-free environment will improve the quality of life

Benefits to Society: The contribution of crop residue burning towards climate change and reduction in soil fertility will be controlled.

**Confidentiality:**

Your right to confidentiality will be respected. No personal information that discloses your identity will be revealed or published without your specific consent for such disclosure. Your identity will be coded and on paper, questionnaires, and transcripts you will be identified by a number (code) only. All information collected as part of this study will be kept confidential and will be used only for research purposes. All information that will be computerized, will be password protected.

**Voluntary Participation and withdrawal:**

Your participation in this study is entirely voluntary. You may refuse to take part or may even decide to withdraw consent to participate at any point in time during the study. If you decide to withdraw from the on-going study then you will have to inform the principal investigator.

However, I request you to choose to participate in this study.

**Whom to contact:** If you have any questions you may ask them now or any time later, even after initiation of the study. **Contact Information:** (Will be provided here)

**PART- 2**

**(CERTIFICATE OF CONSENT)**

**Title of this study:** Community-based participatory action research for situation analysis and mitigation of crop residue burning in Madhya Pradesh, India.

**Name of Principal Investigator:** Dr. Tanwi Trushna, Scientist-B, Epidemiology, ICMR-NIREH, Bhopal

I understand that this is a research study. I have been explained that it is being conducted to understand the different aspects of crop residue burning especially from the perspective of the community and that I will be expected to discuss my opinions and perceptions regarding the practice with the study investigators in interviews and group discussions. I have carefully read and understood the participant information sheet in its entirety. I have been told in detail about the methodology of this project and the steps included therein. I have been guaranteed that I will not have to undergo any invasive procedure nor will I have to bear any monetary expenses for taking part in the study. I have had the opportunity to ask questions to clarify my doubts and all my questions have been answered satisfactorily. I have also been ensured that if I have any further questions in the future then the research investigators will willingly answer them. I have been informed clearly that my participation in this study is completely voluntary and that even after initially consenting to participate, I still retain the autonomy to refuse participation or to withdraw my consent any time during the study process without fear of any adverse consequences.

Having given enough time to deliberate and decide whether or not to participate in this study, I voluntarily, without any undue coercion, decide to give my consent for participation in this study. I hereby authorise the release of data collected from me during interviews and focus group discussions for submission to regulatory authorities, ethics committee, and other scientific forums for the sole purpose of research. I understand that I will be informed of any clinical finding is reported after the study. My signature below indicates that I voluntarily agree to take part in this study.

Parent Name: ………………………………………….. Signature/Thumb impression: ………………..…

Date: ………/…………./……………… Place: ………………………………………………………

Witness Name: ………………………………………………. Signature: ………………………………………………..

Date: ………/…………./……………… Place: ………………………………………………………

Witness Name: ………………………………………………. Signature: ………………………………………………..

Date: ………/…………./……………… Place: ………………………………………………………

Investigator’s Name: ……………………………………… Signature: ………………………………………………..

Date: ………/…………./……………… Place: ………………………………………………………

**Appendix 4: Patient Information Sheet and Consent form- PFT (adults)**

**INFORMED CONSENT FORM**

This informed consent has two parts:

Part-1: Information Sheet (To share information about the study with you)

Part-2: Certificate of Consent (For signature if you agree to take part)

**PART-1**

**(PARTICIPANT INFORMATION SHEET)**

**Title of this study: Community-based participatory action research for situation analysis and mitigation of crop residue burning in Madhya Pradesh, India.**

**Name of Principal Investigator:** Dr. Tanwi Trushna, Scientist-B, Epidemiology, ICMR-NIREH, Bhopal

**Co-Investigators:** Dr. Yogesh Sabde, Dr. Vishal Diwan, Dr. Subroto Nandi, Dr. Satish B. Aher.

**Introduction:**

You are invited to take part in this study. The title of this study is “Community-based participatory action research for situation analysis and mitigation of crop residue burning in Madhya Pradesh, India”. The purpose of this form is to provide you with information regarding this research project and if you agree to sign the consent form, then it means that you are willing to participate. It is very important that you carefully read and understand the information provided in this form. This form describes in detail the purpose, procedure, benefits, and risks of the current study. There might be words or processes that you might not be able to understand and in that case, you are encouraged to ask questions to clarify your doubts and the investigators would be willing to answer them. You are at complete liberty to choose to participate in the study or withdraw your participation at any point in time.

**Purpose of the study:** The current study aims to comprehensively understand the various aspects of crop residue burning from the perspective of the community and to estimate the impact of the same on human health, air quality, and soil. The study will attempt to identify the barriers to the adoption of healthy crop residue management behaviour and possible strategies for behaviour change intervention to promote the same.

**Study Procedures:** Presently I will explain in detail all the procedures and in case, you need extra information, please feel free to ask at any time. No procedure will be initiated before you provide consent to participate in the study. You do not have to give your consent or answer any question if you do not feel comfortable due to any reason. The information that you choose to share with the investigators will be completely confidential and your identity will never be disclosed.

**Details of the procedure:** Investigators will collect information regarding the effect of crop residue burning on the functioning of your respiratory system. It will be done using a free of cost, a non-invasive procedure known as Pulmonary Function Testing (PFT). The process of such data collection will tentatively happen over 6 months during which you will be expected to undergo this investigation five times. Each time the process will last for 20-30 minutes during which the technician administering the test will ask you to breathe in and out into a mouthpiece deeply for multiple times.

**Potential risks or side-effects:** None

**Potential benefits:**

Immediate benefits: Diagnosis and awareness of the adverse effect of crop residue burning on pulmonary function.

Delayed benefits: Better health and a smoke-free environment will improve the quality of life

Benefits to Society: The contribution of crop residue burning towards climate change and reduction in soil fertility will be controlled.

**Confidentiality:**

Your right to confidentiality will be respected. No personal information that discloses your identity will be revealed or published without your specific consent for such disclosure. Your identity will be coded and on paper, questionnaires, and transcripts you will be identified by a number (code) only. All information collected as part of this study will be kept confidential and will be used only for research purposes. All information that will be computerized, will be password protected.

**Voluntary Participation and withdrawal:**

Your participation in this study is entirely voluntary. You may refuse to take part or may even decide to withdraw consent to participate at any point in time during the study. If you decide to withdraw from the on-going study then you will have to inform the principal investigator.

However, I request you to choose to participate in this study.

**Whom to contact:** If you have any questions you may ask them now or any time later, even after initiation of the study. **Contact Information:** (Will be provided here)

**PART- 2**

**(CERTIFICATE OF CONSENT)**

**Title of this study:** Community-based participatory action research for situation analysis and mitigation of crop residue burning in Madhya Pradesh, India.

**Name of Principal Investigator:** Dr. Tanwi Trushna, Scientist-B, Epidemiology, ICMR-NIREH, Bhopal

I understand that this is a research study. I have been explained that it is being conducted to understand the effect of crop residue burning on human health especially that of the respiratory system. I have been told that I will be expected to undergo a painless non-invasive investigation during I will be asked to inhale and exhale deeply following the instructions of a trained technician. I have carefully read and understood the participant information sheet in its entirety. I have been told in detail about the methodology of this project and the steps included therein. I have been guaranteed that I will not have to undergo any other invasive procedure nor will I have to bear any monetary expenses for taking part in the study. I have had the opportunity to ask questions to clarify my doubts and all my questions have been answered satisfactorily. I have also been ensured that if I have any further questions in the future then the research investigators will willingly answer them. I have been informed clearly that my participation in this study is completely voluntary and that even after initially consenting to participate, I still retain the autonomy to refuse participation or to withdraw my consent any time during the study process without fear of any adverse consequences.

Having given enough time to deliberate and decide whether or not to participate in this study, I voluntarily, without any undue coercion, decide to give my consent for participation in this study. I hereby authorise the release of data collected from me during pulmonary function testing for submission to regulatory authorities, ethics committee, and other scientific forums for the sole purpose of research. I understand that I will be informed of any clinical finding that is reported after the study. My signature below indicates that I voluntarily agree to take part in this study.

Parent Name: ………………………………………….. Signature/Thumb impression: ………………..…

Date: ………/…………./……………… Place: ………………………………………………………

Witness Name: ………………………………………………. Signature: ………………………………………………..

Date: ………/…………./……………… Place: ………………………………………………………

Witness Name: ………………………………………………. Signature: ………………………………………………..

Date: ………/…………./……………… Place: ………………………………………………………

Investigator’s Name: ……………………………………… Signature: ………………………………………………..

Date: ………/…………./……………… Place: ………………………………………………………

**Appendix 5: Patient Information Sheet, Parental Consent and Child Assent form- PFT (Child)**

**INFORMED CONSENT FORM**

This informed consent has three parts:

Part-1: Information Sheet (To share information about the study with you)

Part-2: Parental Consent Form (For parent’s signature if he/she agrees to allow his/her child to take part)

Part-3: Certificate of Assent (For child’s signature if he/she is willing to participate)

**PART-1**

**(PARTICIPANT INFORMATION SHEET)**

**Title of this study: Community-based participatory action research for situation analysis and mitigation of crop residue burning in Madhya Pradesh, India.**

**Name of Principal Investigator:** Dr. Tanwi Trushna, Scientist-B, Epidemiology, ICMR-NIREH, Bhopal

**Co-Investigators:** Dr. Yogesh Sabde, Dr. Vishal Diwan, Dr. Subroto Nandi, Dr. Satish B. Aher.

**Introduction:**

Your child is invited to take part in this study. The title of this study is “Community-based participatory action research for situation analysis and mitigation of crop residue burning in Madhya Pradesh, India”. The purpose of this form is to provide you with information regarding this research project and if you agree to sign the consent form, then it means that you are willing to let your child participate. Your child will also be expected to provide his assent for participation in this study. It is very important that you and your child carefully read and understand the information provided in this form. This form describes in detail the purpose, procedure, benefits, and risks of the current study. There might be words or processes that you might not be able to understand and in that case, you are encouraged to ask questions to clarify your doubts and the investigators would be willing to answer them. You are at complete liberty to choose to allow your child to participate in the study or withdraw his/her participation at any point in time.

**Purpose of the study:** The current study aims to comprehensively understand the various aspects of crop residue burning from the perspective of the community and to estimate the impact of the same on human health, air quality, and soil. The study will attempt to identify the barriers to the adoption of healthy crop residue management behaviour and possible strategies for behaviour change intervention to promote the same.

**Study Procedures:** Presently I will explain in detail all the procedures and in case, you need extra information, please feel free to ask at any time. No procedure will be initiated before you provide assent for your child’s participation in the study and your child provides his/her consent for participation. You do not have to give your consent or answer any question if you do not feel comfortable due to any reason. The information that you choose to share with the investigators will be completely confidential and your child’s identity will never be disclosed.

**Details of the procedure:** Investigators will collect information regarding the effect of crop residue burning on the functioning of the respiratory system of your child. It will be done using a free of cost, a non-invasive procedure known as Pulmonary Function Testing (PFT). The process of such data collection will tentatively happen over 6 months during which your child will be expected to undergo this investigation five times. Each time the process will last for 20-30 minutes during which the technician administering the test will ask your child to breathe in and out into a mouthpiece deeply for multiple times.

**Potential risks or side-effects:** None

**Potential benefits:**

Immediate benefits: Diagnosis and awareness of the adverse effect of crop residue burning on pulmonary function.

Delayed benefits: Better health and a smoke-free environment will improve the quality of life

Benefits to Society: The contribution of crop residue burning towards climate change and reduction in soil fertility will be controlled.

**Confidentiality:**

Your child’s right to confidentiality will be respected. No personal information that discloses the identity of your child will be revealed or published without your specific consent for such disclosure. Your child’s identity will be coded and on paper, questionnaires, and transcripts he/she will be identified by a number (code) only. All information collected as part of this study will be kept confidential and will be used only for research purposes. All information that will be computerised, will be password protected.

**Voluntary Participation and withdrawal:**

Participation in this study is entirely voluntary. Your child may refuse to take part or may even decide to withdraw consent to participate at any point in time during the study. If your child decides to withdraw from the on-going study then you will have to inform the principal investigator.

However, I request you to choose to participate in this study.

**Whom to contact:** If you or your child have any questions you may ask them now or any time later, even after initiation of the study. **Contact Information:** (Will be provided here)

**PART- 2**

**(PARENTAL CONSENT FORM)**

**Title of this study:** Community-based participatory action research for situation analysis and mitigation of crop residue burning in Madhya Pradesh, India.

**Name of Principal Investigator:** Dr. Tanwi Trushna, Scientist-B, Epidemiology, ICMR-NIREH, Bhopal

I understand that this is a research study. I have been explained that it is being conducted to understand the effect of crop residue burning on human health especially that of the respiratory system. I have been told that my child will be expected to undergo a painless non-invasive investigation thrice and each time he/she will be asked to inhale and exhale deeply following the instructions of a trained technician. I have carefully read and understood the participant information sheet in its entirety. I have been told in detail about the methodology of this project and the steps included therein. I have been guaranteed that my child will not have to undergo any other invasive procedure nor will I have to bear any monetary expenses for his/her participation in the study. I have had the opportunity to ask questions to clarify my doubts and all my questions have been answered satisfactorily. I have also been ensured that if I have any further questions in the future then the research investigators will willingly answer them. I have been informed clearly that my child’s participation in this study is completely voluntary and that even after initially consenting to participate, I still retain the autonomy to refuse participation or to withdraw my consent any time during the study process without fear of any adverse consequences.

Having given enough time to deliberate and decide whether or not to allow my child to participate in this study, I voluntarily, without any undue coercion, decide to give my consent for my child’s participation in this study. I hereby authorise the release of data collected from my child during pulmonary function testing for submission to regulatory authorities, ethics committee, and other scientific forums for the sole purpose of research. I understand that I will be informed of any clinical finding that is reported after the study. My signature below indicates that I voluntarily agree to let my child take part in this study.

Parent Name: ………………………………………….. Signature/Thumb impression: …………………..

Date: ………/…………./……………… Place: ………………………………………………………

Witness Name: ………………………………………………. Signature: ………………………………………………..

Date: ………/…………./……………… Place: ………………………………………………………

Witness Name: ………………………………………………. Signature: ………………………………………………..

Date: ………/…………./……………… Place: ………………………………………………………

Investigator’s Name: ……………………………………… Signature: ………………………………………………..

Date: ………/…………./……………… Place: ………………………………………………………

**PART- 3**

**(CERTIFICATE OF ASSENT [CHILD])**

**Title of this study:** Community-based participatory action research for situation analysis and mitigation of crop residue burning in Madhya Pradesh, India.

**Name of Principal Investigator:** Dr. Tanwi Trushna, Scientist-B, Epidemiology, ICMR-NIREH, Bhopal

I understand that this is a research study. I have been told that I will be expected to undergo a painless non-invasive investigation thrice and each time I will be asked to inhale and exhale deeply following the instructions of a trained technician. I have had the opportunity to ask questions to clarify my doubts and all my questions have been answered satisfactorily. I have also been ensured that if I have any further questions in the future then the research investigators will willingly answer them. I have been informed clearly that my participation in this study is completely voluntary and that even after initially consenting to participate, I still retain the autonomy to refuse participation or to withdraw my consent any time during the study process without fear of any adverse consequences. I agree to participate in this study.

Name of Child: ………………………………………….. Signature/Thumb impression: ………………….

Date: ………/…………./……………… Place: ………………………………………………………

Parent Name: ………………………………………………. Signature: ………………………………………………..

Date: ………/…………./……………… Place: ………………………………………………………

Witness Name: ………………………………………………. Signature: ………………………………………………..

Date: ………/…………./……………… Place: ………………………………………………………

Investigator’s Name: ……………………………………… Signature: ………………………………………………..

Date: ………/…………./……………… Place: ………………………………………………………
